# Associations between Body Mass and the Outcome of Surgery for Scoliosis in Chinese Adults

**DOI:** 10.1371/journal.pone.0021601

**Published:** 2011-07-01

**Authors:** Ziqiang Chen, Honglei Yi, Ming Li, Chuanfeng Wang, Jingtao Zhang, Changwei Yang, Yingchuan Zhao, Yanghu Lu

**Affiliations:** 1 Department of Orthopaedic Surgery, The Affiliated Changhai Hospital of the Second Military Medical University, Shanghai, China; 2 Chinese People's Liberation Army 89 Hospital, Weifang, China; University Medical Center Groningen UMCG, The Netherlands

## Abstract

**Background:**

In this study we intended to prove that being overweight has an unfavorable impact on the surgical treatment outcome of adult idiopathic scoliosis (AdIS).

**Methods:**

This is a retrospective study on the surgical treatment of seventy-one more than 30 years old (58 females and 13 males; mean age 42.9±12.2) idiopathic scoliotic patients with a minimum follow up of at least 2 years. The patients were divided into an overweight group (BMI≥23) and a non-overweight group (BMI<23). Preoperative, postoperative first erect and final follow-up radiographic measures, perioperative data, the Oswestry disability index (ODI), and the visual analog scale (VAS) were reviewed and compared.

**Findings:**

In the overweight group, no significant differences in radiographic measures, perioperative data, preoperative comorbidities, or postoperative complications, except for the more frequent concomitance of preoperative thoracic kyphosis 37.9±7.7 vs. 26.5±11.8 (*P* = 0.000) and thoracolumbar kyphosis 14.9±10.1 overweighted group vs. 6.5±9.9 non-overweighted group respectively (*P* = 0.002) were found. A higher morbidity of hypertension 36.8% vs. 9.6% (*P* = 0.004) was also observed in the overweight group. Postoperative ODI and VAS improved significantly in both groups compared to pre-operative values. The postoperative ODI of the overweight group (19.6±12.4) was significantly higher than that of the non-overweight group (12.4±7.9) (*P* = 0.022).

**Conclusions:**

Overweight adult idiopathic scoliotic patients had more frequent concomitance of preoperative thoracic kyphosis and thoracolumbar kyphosis and more serious postoperative pain. However, BMI did not affect the outcomes of surgical correction for coronal and sagittal scoliotic deformity and their postoperative complication rates were not significantly affected.

## Introduction

According to statistics from the World Health Organization (WHO), obesity has reached epidemic proportions; more than one billion adults are now classified as being overweighted, and at least 300 million of these adults are classified as clinically obese. Obesity is a major contributor to the global burden of chronic disease and disability. With improvements in the living standards of Chinese people and changes in their dietary structure, the incidence of obesity is on the rise in China [1]. Obesity contributes to numerous non-communicable chronic diseases and leads to more healthcare expenditures than any other medical condition. Body mass index (BMI), an index calculated using one's weight and height, is a reliable indicator of body fatness for most people, and it has been used to screen weight categories that may lead to health problems. It has been used by the National Institutes of Health to categorize patients based on their health risks [2]. The greater a person's BMI, the greater their risk of comorbidities, including diabetes mellitus, hypertension, cancers, dyslipidemia, and cardiovascular diseases, and the greater their overall mortality. Adult obesity has also been associated with an increased rate of postoperative surgical complications [3].

Although there have been many studies on the associations between obesity and other chronic illnesses, the association of obesity with scoliosis has not been intensively studied. A study on the effect of BMI on the surgical outcome of adolescent idiopathic scoliosis (AIS) did not identify any associations between BMI and surgical outcomes of AIS [4]. The authors attributed this result to comorbidities that were responsible for the increased perioperative complications in their adult population rather than in the adolescent cohort. We also observed that obesity-induced complications were not evident before adulthood. In addition, the effect of body weight on the vertebrae and the intervertebral elements increases with age. Surveys about obesity indicate that BMI in adulthood increases with age, especially after 30 years of age. The result of a large-sample epidemic survey in Chinese adults showed that there was a significant difference in the proportion of overweight individuals between those who were younger than 30 years and those who were older than 30 years [2]. To avoid a possible age bias between overweight and non-overweight groups and due to the fact that the pathologic features of adult idiopathic scoliosis (AdIS) in patients younger than 30 years are similar to those of AIS, the present study only included adult idiopathic scoliotic patients older than 30 years of age. In summary, overweight increases the stress load on the body and accelerates spinal degeneration with age. In this retrospective study we investigated the influence of obesity on orthopedic treatment of scoliosis in patients older than 30 years. Additionally, preoperative comorbidities accompanying with over-weight, which are more common in adults than in adolescents, may increase perioperative complications. As a result we could not observe that the BMI had an effect on the outcomes of surgical corrections for coronal and sagittal scoliotic deformity. Although overweight patients were more likely to have pre-existing hypertension, their postoperative complication rates were not significantly affected.

## Methods

### Objectives

In this retrospective analysis we investigated the effect of obesity on outcomes of surgical scoliosis treatments, because overweight is a general stress factor for joints and is leading to accelerated spinal degeneration with age. Furthermore, preoperative comorbidities related with over-weight are more common in adults than in adolescents and we thought they might increase perioperative complications as well as having an complicating impact on the outcome of scoliosis surgery in patients older than 30 years.

### Participants

Patients older than 30 years, who sought medical help due to AdIS in our hospital between July 2004 and April 2007 and had been followed up for at least two years after their surgical treatment, were retrospectively reviewed. The diagnosis of AdIS followed the Aebi classification system of adult scoliosis [5]. Other subtypes of scoliosis were excluded by medical history, physical examination, and full-spine MRI examination, especially adult degenerative scoliosis, which mainly occurs in middle-aged and older patients in the absence of pre-existing scoliosis. Adult degenerative scoliosis mainly involves the thoracolumbar and lumbar segments and ususlly has angles less than 60°. It has a primary symptom of lower back pain and leg pain associated with spinal stenosis. The diagnosis was made independently by three attending clinicians, and only patients who were diagnosed with AdIS by all the three doctors were included in the present study. Anthropometric measurements included height and weight. Corrected height was derived from Bjure's formula (log y = 0.011x−0.177), where y is the loss of trunk height (cm) due to the deformed spine and x is the greatest Cobb's angle of the primary curve [6]. BMI was determined by dividing the weight (kg) by the square of the corrected height (m^2^). In asians the WHO changed the criteria for overweight of BMI≥25, applied for western people to BMI≥23. This reduction by 2 index values as overweight limit was introduced in order to avoid the wrong estimate of low prevalence of overweight in asians with yet high rates of obesity-related diseases. Using the WHO classification for Asian populations, with a reduced BMI limit of ≥ 23[7] [8], the patients in this cohort were divided into two groups: non-overweight (BMI<23) and overweight (BMI≥23).

### Surgical procedures

All scoliotic patients in our hospital received traction with suspended weights and respiratory training before surgery. Operations for all patients were performed by the same orthopedist team through a posterior approach. Intraoperative spinal cord monitoring was routinely performed. The spinal fixation systems used in this study were third-generation system rods and screws without any hook, including Expedium™ (DePuy Spine, Raynham, MA) in 22 patients, Moss® Miami (DePuy Spine, Raynham, MA) in 39 patients, CD Horizon®Legacy™ (Medtronic, Minneapolis, MN) in 7 patients, and Xia (Stryker, Kalamazoo, MI) in 3 patients. After the implantation of orthopedic rods, the spinal processes were resected and the vertebral plates were finished. The homogeneity-variant bone grafts and autologous bone grafts were implanted to enhance fusion without using bone morphogenetic proteins. Patients were instructed to wear braces for 3 months after surgery.

### Surgical and Follow-up Outcome Measures

Primary surgical and follow-up outcome measures consisted mainly of the Oswestry disability index (ODI) [9] and the visual analog scale (VAS) for lower back and leg pain [10]. ODI was a self-assessment questionnaire with a 10-item scale; scores of 0 to 5 were given for each variable and then transformed into a higher-worse scale of 0-100. The severity of disability was categorized as follows: 0–20, minimal disability; 21–40, moderate; 41–60, severe; 61–80, crippled; and 81–100, bed-ridden or with exaggerated symptoms. The pain VAS was a two-item numerical rating scale from 0 (no discomfort or pain) to 10 (unbearable pain) for both lower back pain and leg pain. In addition, the standing posterior anterior and lateral full-length spinal radiographs were captured at the postoperative follow-up visits at 3, 6, 12, and 24 months after surgery.

### Patients and Anthropometric Measurements

The study protocol was approved by the Institutional Review Board of the Second Military Medical University, Shanghai, China. Written informed consent was obtained from every participant. The protocols of this study have been approved by the institutional review board of Changzheng Hospital.

### Statistical Analysis

Patients were excluded from subsequent analysis unless they had complete documentation of their surgical and follow-up data, including perioperative and radiographic data, preoperative comorbidities, postoperative complications, and ODI and pain VAS at baseline, as well as two years of follow-up visits. An independent Student's t-test of variance and the Chi-square test were used to compare the differences between dependent variables in the two groups over time. Data were checked for the normality and equal variances. For each individual analysis within the study, a P value less than 0.05 was considered significant. Statistical Package for Social Science (SPSS) software (SPSS Inc., Chicago, IL) was used to perform the statistical analysis.

## Results

### Baseline and Perioperative Characteristics of Patients

This study included 71 AdIS patients (58 females and 13 males; aged 30.1–77.5 yr, mean 42.9±12.2 yr). Their mean weight was 56.3±7.1 kg (range of 42.2–72.1 kg) and their mean height was 1.64±0.06 m (range of 1.49–1.77 m). The mean BMI for this cohort was 21.7±2.4 kg/m^2^ (range of 15.7–28.0 kg/m^2^). Table 1 shows the descriptive characteristics of the two groups, including both overweight (n = 19) and non-overweight (n = 52) patients. There was no significant difference in age, age of onset, or gender ratio. There was no significant difference in perioperative data, including surgical approaches, operating time, estimated volume of blood loss, duration of hospital stay, cost of operation, and fusion level between the two groups (Table 2).

**Table 1:** Summary of Demographics and Baseline Characteristics of Adult Idiopathic Scoliotic Patients.

Variable	Overweight, Mean ± SD (n = 19)	Non-overweight, Mean ± SD (n = 52)	*P*
Age (year)	46.0±14.5	41.8±11.2	0.196
Age of onset (year)	17.1±8.1	16.0±4.9	0.521
Male: Female	3: 16	10: 42	0.737
Corrected height (m)	1.62±0.04	1.65±0.06	0.072
Weight (kg)	62.8±5.5	53.9±6.2	0.000
BMI (kg/m^2^)	24.6±1.6	20.1±1.6	0.000

SD indicates standard deviation; BMI, body mass index.

**Table 2:** Surgical Outcomes of Adult Idiopathic Scoliotic Patients.

Surgical outcomes		Overweight, Mean ± SD (n = 19)	Non-overweight, Mean ± SD (n = 52)	*P*
Surgical approach (n, %)	Posterior only	17 (89.55)	41 (78.9%)	0.369
	Anterior and posterior	1 (5.3%)	2 (3.85%)	
	Posterior and thoracoplasty	1 (5.3%)	9 (17.3%)	
Operation time (min)		219.5±59.5	241.7±62.6	0.185
Estimated blood loss (mL)		1684.2±932.9	1676.9±747.7	0.667
Length of hospital stay (day)		22.2±6.4	23.4±6.7	0.496
Fusion level (n)		9.9±3.0	10.5±2.7	0.492
Financial burden (RMB)		140871.3±40401.4	142763.1±38326.3	0.857

SD indicates standard deviation.

### Radiographic Outcomes

**Figure 1:**
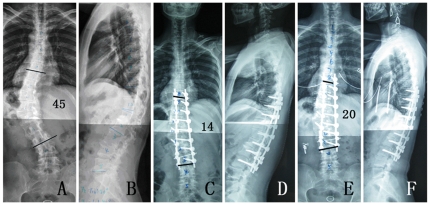
41-year-old female with a BMI of 27.7 diagnosed with thoracolumbar idiopathic scoliosis. (A) anterior-posterior standing and (B) standing lateral full-length spinal preoperative X-rays showing coronal imbalance and sagittal thoracolumbar kyphosis; (C) anterior-posterior standing and (D) standing lateral full-length spinal X-rays: the first post-surgical erect X-rays showing the correction and fusion; (E) anterior-posterior standing and (F) standing lateral full-length spinal X-rays: the two-year follow-up X-rays showed a good balance.

With respect to the preoperative (Figs. 1A, B), postoperative first erect (Figs. 1C, D), and final follow-up (Figs. 1E, F) radiographic parameters, only preoperative thoracic kyphosis and thoracolumbar kyphosis were more frequent in the overweight group than in the non-overweight group (*P = *0.000 and *P = *0.002, respectively) (Figs. 1A, B). No significant difference was observed in any other radiographic parameters (Tables 3, 4, 5).

**Table 3:** Preoperative Radiographic Measures of Adult Idiopathic Scoliotic Patients.

Radiographic measures		Overweight, Mean ± SD (n = 19)	Non-overweight, Mean ± SD (n = 52)	*P*
Cobb's angle (°)		55.1±16.5	60.7±18.3	0.248
Primary curve (n, %)	Thoracic	10 (77.8%)	38 (83.6%)	0.103
	Lumbar	9 (22.2%)	14 (16.4%)	
Flexibility		0.40±0.20	0.33±0.15	0.169
Thoracic kyphosis (°)		37.9±7.7	26.5±11.8	0.000
Thoracolumbar kyphosis (°)		14.9±10.1	6.5±9.9	0.002
Lumbar lordosis (°)		31.3±13.7	31.4±14.2	0.985
Coronal C7 CSVL distance (cm)		2.1±1.3	1.7±1.1	0.261
Apical translation from CSVL (cm)		5.3±1.2	5.5±1.9	0.588
Maximal lumbar translation (cm)		0.9±1.1	0.8±0.8	0.561
Pelvic tilt (°)		1.8±2.2	1.2±1.5	0.143
Lateral C7 to CSVL distance (cm)		−0.3±2.5	−1.2±3.1	0.242
Maximal lumbar olisthy (cm)		0.3±0.3	0.2±0.3	0.336

SD indicates standard deviation; CSVL, center sacral vertical line.

**Table 4:** Postoperative Radiographic Measures of Adult Idiopathic Scoliotic Patients.

Radiographic measures	Overweight, Mean ± SD (n = 19)	Non-overweight, Mean ± SD (n = 52)	*P*
Cobb's angle (°)	27.3±14.1	31.1±17.9	0.403
Correction rate (%)	52.6±13.9	51.8±16.4	0.840
Thoracic kyphosis (°)	30.7±7.1	28.2±6.0	0.150
Thoracolumbar kyphosis (°)	8.2±8.6	5.9±7.6	0.272
Lumbar lordosis (°)	34.9±7.1	28.2±6.0	0.356
Coronal C7 CSVL distance (cm)	1.4±1.4	1.3±1.1	0.835
Apical translation from CSVL (cm)	2.7±1.2	3.0±1.9	0.487
Maximal translation of lumbar (cm)	0.4±0.7	0.4±0.6	0.985
Pelvic tilt (°)	1.0±1.0	0.8±1.4	0.494
Lateral C7 to CSVL distance (cm)	-0.2±2.0	-1.0±2.3	0.154
Maximal lumbar olisthy (cm)	0.1±0.3	0.1±0.3	0.154

SD indicates standard deviation; CSVL, center sacral vertical line.

**Table 5:** Final Follow-up Radiographic Measures of Adult Idiopathic Scoliotic Patients.

Radiographic measures	Overweight, Mean ± SD (n = 19)	Non-overweight, Mean ± SD (n = 52)	*P*
Cobb's angle (°)	30.0±13.6	33.7±18.1	0.407
Cobb's angle decreased (°)	2.8±2.3	2.8±2.7	0.977
Correction rate (%)	47.0±12.9	47.0±15.9	0.986
Thoracic kyphosis (°)	31.4±6.2	29.5±6.4	0.247
Thoracolumbar kyphosis (°)	5.4±4.3	4.5±5.3	0.506
Lumbar lordosis (°)	36.4±6.1	34.6±6.5	0.312
Coronal C7 CSVL distance (cm)	1.1±0.4	1.1±0.7	0.950
Apical translation from CSVL (cm)	2.3±1.0	2.7±1.6	0.266
Maximal translation of lumbar (cm)	0.4±0.5	0.4±0.5	0.969
Pelvic tilt (°)	0.7±0.7	0.5±0.9	0.356
Lateral C7 to CSVL distance (cm)	−1.0±1.9	−1.2±1.7	0.679
Maximal lumbar olisthy (cm)	0.2±0.3	0.1±0.2	0.311

SD indicates standard deviation; CSVL, center sacral vertical line.

### Symptom Outcomes

With respect to symptoms, there was no significant difference in preoperative ODI and VAS between the two groups. Both ODI and VAS were improved significantly in both groups compared to pre-operative values (overweight group: *P = *0.008 for ODI and *P = *0.027 for VAS; non-overweight group: *P = *0.000 for ODI and *P = *0.000 for VAS). The postoperative ODI in the overweight group was significantly higher than in the non-overweight group (*P = *0.022), but the postoperative VAS in the overweight group did not differ significantly from that in the non-overweight group (*P = *0.092) (Table 6).

**Table 6:** Clinical Improvements in Lower Back and Leg Pain Syndrome of Adult Idiopathic Scoliotic Patients.

Variable	Overweight, Mean ± SD (n = 19)	Non-overweight, Mean ± SD (n = 52)	*P*
Preoperative ODI	27.1±23.1	19.8±19.3	0.184
Two-year follow-up ODI	19.6±12.4	12.4±7.9	0.022
Preoperative VAS	3.6±2.4	3.1±2.3	0.430
Two-year follow-up VAS	3.5±1.6	2.6±1.4	0.092

SD indicates standard deviation; ODI, Oswestry disability index; VAS, visual analogue scale.

### Concomitant Morbidity Outcomes

With respect to preoperative morbidity, including hypertension, diabetes mellitus, and coronary heart disease, the occurrence of hypertension in the overweight group was significantly more frequent than in the non-overweight group (*P = *0.004), whereas the occurrence of the other two complications did not differ significantly between the two groups (*P = *0.082 for diabetes mellitus; *P = * 0.111 for coronary heart disease) (Table 7). There was no significant difference in postoperative complications between the two groups (*P = *0.069 for surgical site infection; *P = *0.096 for adjacent segment disease) (Table 8).

**Table 7:** Pre-existing Comorbidities of Adult Idiopathic Scoliotic Patients.

Comorbidities	Overweight, (n = 19)	Non-overweight, (n = 52)	*P*
Hypertension (n, %)	7 (36.8%)	5 (9.6%)	0.004
Diabetes mellitus (n, %)	3 (15.8%)	2 (3.8%)	0.082
Coronary heart disease (n, %)	2 (10.5%)	1 (1.9%)	0.111

**Table 8:** Procedure-related Complications of Adult Idiopathic Scoliotic Patients.

Complications	Overweight, (n = 19)	Non-overweight, (n = 52)	*P*
Pneumonia (n, %)	1 (5.3%)	0 (0.0%)	0.268
Pulmonary embolism (n, %)	0 (0.0%)	0 (0.0%)	-
Surgical site infection (n, %)	2 (10.5%)	0 (0.0%)	0.069
Neurologic deficit (n, %)	0 (0.0%)	0 (0.0%)	-
Cerebrospinal fluid leakage (n, %)	1 (5.3%)	0 (0.0%)	0.268
Urinary tract infection (n, %)	0 (0.0%)	1 (1.9%)	1.000
Pseudarthrosis (n, %)	2 (10.5%)	3 (5.8%)	0.865
Surgical revision (n, %)	1 (5.3%)	0 (0.0%)	0.268
Ileus (n, %)	1 (5.3%)	2 (3.8%)	1.000
Adjacent segment disease (n, %)	3 (15.8%)	1 (1.9%)	0.096
Transient delirium (n, %)	0 (0.0%)	1 (0.0%)	1.000
Loosening of screws (n, %)	1 (5.3%)	0 (0.0%)	0.268

## Discussion

Adult scoliosis is defined as spinal curvature in the coronal plane greater than 10° in a skeletally mature spine. According to the Aebi classification [5], AdIS is idiopathic adolescent scoliosis of the thoracic and/or lumbar spine that progresses throughout adulthood and is usually associated with secondary degeneration and/or imbalance. Most previous studies on AdIS failed to discriminate adult idiopathic scoliosis from degenerative scoliosis, although the two subtypes differ significantly in their causes and features. The latter disorder occurs more frequently in middle-aged and older patients and has less severe involvement of the thoracolumbar and lumbar segments than AdIS. The primary symptom of degenerative scoliosis is lower back and leg pain associated with spinal stenosis.

AdIS patients first exhibit scoliosis during adolescence and occasionally during young adulthood (under 40 years old). The primary curvature usually involves mainly thoracic vertebrae. The curvature can be Lenke types 1–6. AdIS patients seek orthopedic treatment due to progression of deformed curvature in addition to occasional secondary lower back and leg pain. Weinstein et al. [11] emphasized the role of Cobb's angle in predicting the progression of idiopathic scoliosis at maturity through a long-term follow-up study. Their study showed that no scoliotic curve of any subtype worsens in patients with Cobb's angle <30°, but in those with Cobb's angle ≥30°, especially in those with thoracoscoliosis of 50–70°, scoliotic curvature worsens at a rate of 0.75–1° per year. Different subtype of scoliosis also contributes to the prediction of curve progression. At maturity, thoracic scoliosis has the largest Cobb's angle, followed by those with double primary curves at maturity, thoracolumbar scoliosis, and lumbar scoliosis. Additionally, mature thoracolumbar scoliosis has the most rapid progression, whereas in the case of lumbar scoliosis, the right scoliosis doubles the progression rate of the left counterpart. Some AdIS patients seek surgical correction in adulthood due to the progression of curvature used to be mild during adolescence but has increased during adulthood, dissatisfaction with deformed physical appearance, and lower back or leg pain. Furthermore, in developing countries like China, many patients are unable to receive surgical intervention when they are young because of either financial or medical disadvantages, but the same patients can afford corrective surgery at adulthood. With the aging population, AdIS is becoming an increasingly prevalent pathologic entity of the spine. Patients afflicted with this condition usually present with increasing back pain and progressive curvature. Given the wide variety of comorbidities in this elderly patient population, there is no single ideal treatment. Interventions should be adjusted according to the individual patient's conditions and expectations.

As the number of overweight individuals in the population continues to grow, it is important to understand the effect of obesity on the surgical outcomes of orthopedic procedures. These patients' increased body mass is thought to compromise the ability of the spine to be effectively molded in the orthotic treatment of scoliosis. The increased mass is also associated with higher preoperative comorbidity and a higher risk of postoperative complications. However, the association between obesity and surgical outcomes has not been studied in adult scoliotic populations. This was the first study to examine the effect of BMI on AdIS. Our results showed that body mass did not affect the surgical outcome of correction for coronal or sagittal deformities. However, we found that patients with a higher BMI were more likely to have preoperative thoracic/thoracolumbar kyphosis, which may be due to the increased load on the spine with increasing BMI, thus increasing the risk of kyphosis. In addition, pre-existing osteoporosis in some elderly patients might cause minor compression fractures in the anterior edge of the vertebra, which could also increase the risk of thoracic/thoracolumbar kyphosis. Upasani et al. [4] reported that preoperative thoracic kyphosis was significantly greater in the overweight group. They argued that the increased truncal mass might exert a compressive force on the anterior vertebral growth plates, restricting the “anterior overgrowth” effect in adolescent idiopathic scoliosis [12], [13], [14], [15], [16], [17]. The results of our study in an adult population might be due to the “legacy effect” from the pathogenesis during adolescence.

Pain rather than deformity is the main cause that urges patients and orthopedists to replace conservative treatment of AdIS with surgical intervention [18]. In a study on lumbar spine fusion, Djurasovic et al. [19] found that there was no significant difference in the mean improvement between obese patients and non-obese patients with respect to back pain, leg pain, Short Form (36) Health Survey Physical Component Summary (SF-36 PCS) score, or ODI score. However, both SF-36 score (*P = *0.037) and ODI score (*P = *0.028) were better in non-obese patients than in obese patients during our 2-year follow-up period. In our study, we found that there was no significant difference in preoperative ODI and VAS between the two groups. However, a 2-year postoperative follow-up study showed that ODI was higher in the overweight group.

Increased body weight increases the load on the spine and the paraspinal muscles, causing the pain caused by the imbalance in paraspinal muscle tension to become more pronounced. In addition, the increased stress of the adjacent segment intervertebral disks and the secondary degeneration of the intervertebral disk may also contribute to the increased pain. The incidence of adjacent segment disease in the overweight group did not differ significantly from the incidence in the overweight group (15.8% vs. 1.9%, *P = *0.096) in our study. Hangai et al. [20] pointed out that a higher BMI was associated with disk degeneration of L2/3 (odds ratio [OR], 2.98), L3/4 (OR, 3.58), L4/5 (OR, 2.32), and L5/S1 (OR, 3.34). In our study, symptomatic adjacent segment disease is defined as the recurrence of lower back pain and/or the radiating pain to the legs that persists for more than 6 weeks in spite of conservative treatment. In addition, radiographic abnormalities corresponding to clinical symptoms need to be identified. Patients who demonstrated asymptomatic radiographic abnormalities were excluded. The strict definition and short-term follow-up might have limited our ability to detect significant differences between the two groups in the present study. The symptoms from degeneration of adjacent segments of intervertebral disks might not be evident within the 2-year period. This difference might become clear with a longer follow-up period.

The subcutaneous fat in overweight patients is relatively thicker; thus, more tissues are exposed during surgery. The deep soft tissue envelope may increase intraoperative blood loss and make some surgical procedures more challenging. Because operating time may be longer and the volume of intraoperative blood loss may be greater in obese patients, a preoperative assessment and preparations for transfusion should be made. However, in the present study, there was no significant difference in surgical time and intraoperative blood loss between the overweight and non-overweight groups as long as their surgical modalities and fusion segments were similar. Whether this result was due to our small sample size or multiple factors is unknown.

Our study showed that the preoperative incidence of hypertension in the overweight group was higher than that in the non-overweight group, whereas the incidence of diabetes and coronary heart disease in the overweight group did not differ significantly from the non-overweight group. It was presumed that the concomitance of multiple preoperative comorbidities might increase difficulties with anesthesia and postoperative complications. However, Patel et al. [21] found that the complication rate was independent of the presence of diabetes and hypertension. In their 84-case study on the association of spinal surgery complications with obesity, they revealed that obesity might increase the prevalence of perioperative complications as a whole, but occurrences of minor complications increased with age rather than BMI. Another study on lumbar fusion showed that the overall complication rate was slightly higher in the obese group, mainly because of wound-related problems [3]. In our study, there was no significant difference in postoperative complications between the two groups. The postoperative infection rate of the overweight group did not differ significantly from the non-overweight group (10.5% vs. 0%, *P = *0.065). More subcutaneous fat in overweight patients may also contribute to increased susceptibility to infection.

The results of the present study might be restricted by the limited sample size caused by our strict inclusion criteria. A study that includes more AdIS patients for a longer follow-up period is required to further evaluate the subtle differences in perioperative morbidities in the obese population.

In conclusion, overweight adults with scoliosis had a greater morbidity of preoperative thoracic kyphosis and thoracolumbar kyphosis than non-overweight patients. Additionally, more adipose patients were prone to postoperative pain, and being overweighted increased the risk of pre-existing hypertension. However, BMI did not influence the results of coronal and sagittal scoliotic deformity correction.
